# Supplementary material for: Splice-Junction-Based Mapping of Alternative Isoforms in the Human Proteome
Source: Cell Rep. Author manuscript; Available in PMC 2020 Jan 15. (PMC6961840; doi:10.1016/j.celrep.2019.11.026)
Supplement: 2 [file NIHMS1546469-supplement-2.zip › DF1/PXD006675/MitralValve/MitralValve_1_CLTCL1_LQEHFQLQNLGINPANIGFSTLTMESDK.pdf]

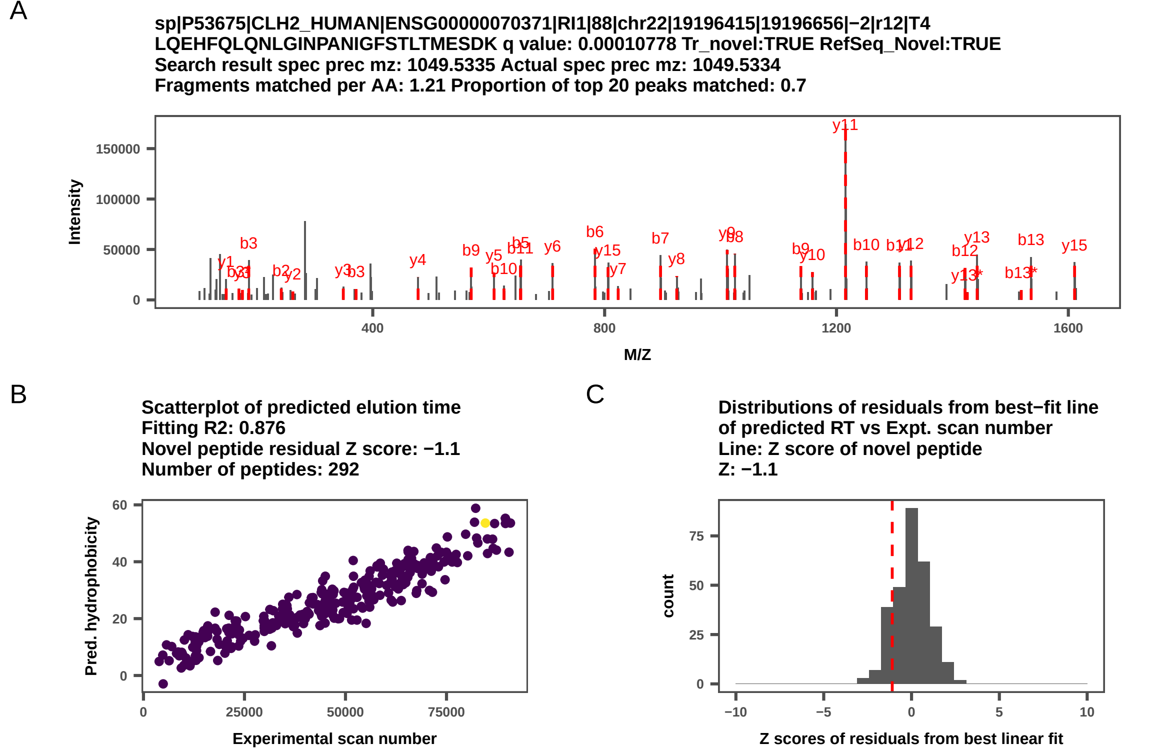
A
sp|P53675|CLH2_HUMAN|ENSG00000070371|RI1|88|chr22|19196415|19196656|−2|r12|T4
LQEHFQLQNLGINPANIGFSTLTMESDK q value: 0.00010778 Tr_novel:TRUE RefSeq_Novel:TRUE
Search result spec prec mz: 1049.5335 Actual spec prec mz: 1049.5334
Fragments matched per AA: 1.21 Proportion of top 20 peaks matched: 0.7
Intensity
150000
100000
50000
0
M/Z
400
800
1200
1600
y11
b3
y1
y2
y3
b3
y4
b9
y5
b5
b11
b10
y6
b6
y15
y7
b7
y8
y9
y8
b9
y10
b10
y12
b12
y13
b13
y15
B
Scatterplot of predicted elution time
Fitting R2: 0.876
Novel peptide residual Z score: −1.1
Number of peptides: 292
Pred. hydrophobicity
60
40
20
0
Experimental scan number
0
25000
50000
75000
C
Distributions of residuals from best−fit line
of predicted RT vs Expt. scan number
Line: Z score of novel peptide
Z: −1.1
count
75
50
25
0
Z scores of residuals from best linear fit
−10
−5
0
5
10
